# Are cataract surgery campaigns necessary for preventing blindness in developing countries?

**DOI:** 10.5935/0004-2749.2024-1016

**Published:** 2025-06-09

**Authors:** Newton Kara-Junior

**Affiliations:** 1 Ophthalmology Department, Universidade de São Paulo, São Paulo, SP, Brazil; 2 Editor-in-Chief of Arquivos Brasileiros de Oftalmologia, São Paulo, SP, Brazil

Cataract campaigns began in the 1980s as a strategy to prevent blindness, because cataracts are one of the main causes of preventable blindness in Brazil and worldwide^(1)^. In 1986, Prof. Newton Kara José developed the first cataract campaign, called the Cataract Project, to prevent blindness in Brazil. It was an active search strategy, in which people with cataract-related blindness were diagnosed during the campaign and later scheduled to undergo routine surgery at university hospitals^(2)^.

The World Health Organization (WHO) has estimated that to offset the annual demand for people in Brazil who develop cataract-related blindness, at least 600,000 surgeries per year would need to be performed. As only 25% of the population has access to private medicine, 450,000 surgeries would be need to be performed per year in the public health system (*Sistema Único de Saúde*, SUS). Furthermore, to compensate for economic blindness and prevent people from abandoning their professions due to poor vision, 1 million procedures would need to be performed, which represents 750,000 surgeries per year in the SUS. However, this estimate does not consider the visual rehabilitation of patients accumulated over time^(3)^.

An analysis of the SUS database over the years has revealed that only 130,000 cataract surgeries were performed annually at the beginning of the 2000s. In 2001, the National Cataract Campaign (CNC) was implemented by the Ministry of Health. In this campaign, the increase in the number of surgeries was stimulated by “extra ceiling” funding from the federal government^(4^-^7)^. With the CNC, the Cataract Project gained national coverage and all diagnosed cases of cataract-related blindness were routinely operated on in public hospitals throughout the country. The guarantee of financing motivated the in-crease in the surgical capacity of the hospitals^(8)^.

In 2003, 430,000 cataract surgeries were performed in the SUS, demonstrating that the determining factor in preventing cataract-related blindness in Brazil was financing^(9)^. In 2006, the Ministry of Health discontinued the CNC. In the subsequent years, the number of cataract surgeries performed in the SUS decreased. A rate of 430,000 annual procedures was achieved again only in 2011, when a new public policy was instituted. In this policy, the surgeries were directly contracted by the government via tenders from companies and private institutions. In 2013, 530,000 procedures were performed in the SUS, and for the first time, the minimum number of surgeries necessary to avoid the accumulation of people with cataract-related blindness was achieved^(10)^.

At the beginning of the 2020s, one of the main sources of financing for cataract surgeries in the SUS were parliamentary amendments, directed to specific municipalities, via Social Health Organizations’ (OSS) strategy to combat blindness. This proved to be effective in increasing the number of surgeries performed, with approximately 860,000 cataract surgeries being performed in 2023. However, the following two limitations were observed: (i) the choice of the investment location was defined by the parliamentarian without considering national public health priorities; and (ii) surgeries were performed in large quantities in a short period of time in adapted structures^(11)^.

Recently, the media has presented the collective efforts taken throughout Brazil. Some have presented the results that were not those sought and desired by people expecting better and the involved professionals who aimed to meet these expectations. However, these surgeries have been performed in adapted surgical centers^(12)^.

Dozens of people have experienced postoperative complications, with several of them losing their eyes due to infections. The majority of these people were affected by endophthalmitis, which is a rare infection. In safe hospital conditions, when the necessary protocols are followed, the incidence of endophthalmitis is approximately 1 in every 1,000 procedures. However, the serial occurrence of this infection indicates that the hospital condition is concerning^(13)^.

Currently, campaigns for carrying out surgeries do not resemble the initial proposal for campaigns for diagnosing and distributing confirmed cases to be routinely operated on in hospitals. Furthermore, for the first time in history, the public health system has performed a sufficient number of cataract surgeries to compensate for the accumulation of new cases and reduce the number of people with cataract-related blindness. However, there are questions regarding the appropriateness of the methods.

The history of cataract surgeries and campaigns demonstrates that the safest alternative would be to maintain the initial proposal of carrying out campaigns to diagnose patients with cataract and scheduling routine surgeries in the regional hospitals, while avoiding the unnecessary overloading of surgeons and security protocols. In this scenario, the logistics would entail the facilitation of patient transport and expansion of the surgical capacity of the regional hospitals. This would engage them in the project, which has been proven to be feasible by the CNC. Despite the greater visibility and practicality of swiftly resolving the problem with this single step, there is no justification for hundreds of surgeries to be performed by a few doctors in a short timeframe and in adapted surgical centers.
